# Effects of low temperature plasmas and plasma activated waters on *Arabidopsis thaliana* germination and growth

**DOI:** 10.1371/journal.pone.0195512

**Published:** 2018-04-09

**Authors:** Maxime Bafoil, Achraf Jemmat, Yves Martinez, Nofel Merbahi, Olivier Eichwald, Christophe Dunand, Mohammed Yousfi

**Affiliations:** 1 LAPLACE, UMR CNRS 5213, Université Paul Sabatier, Toulouse, France; 2 Laboratoire de Recherche en Sciences Végétales, Université de Toulouse, CNRS, UPS, Auzeville, Castanet Tolosan, France; 3 Fédération de Recherche 3450, Plateforme Imagerie, Pôle de Biotechnologie Végétale, Castanet-Tolosan, France; University of Massachusetts Amherst, UNITED STATES

## Abstract

Two plasma devices at atmospheric pressure (air dielectric barrier discharge and helium plasma jet) have been used to study the early germination of *Arabidopsis thaliana* seeds during the first days. Then, plasma activated waters are used during the later stage of plant development and growth until 42 days. The effects on both testa and endospserm ruptures during the germination stage are significant in the case of air plasma due to its higher energy and efficiency of producing reactive oxygen species than the case of helium plasma. The latter has shown distinct effects only for testa rupture. Analysis of germination stimulations are based on specific stainings for reactive oxygen species production, peroxidase activity and also membrane permeability tests. Furthermore, scanning electron microscopy (SEM) has shown a smoother seed surface for air plasma treated seeds that can explain the plasma induced-germination. During the growth stage, plants were watered using 4 kinds of water (tap and deionized waters activated or not by the low temperature plasma jet). With regards to other water kinds, the characterization of the tap water has shown a larger conductivity, acidity and concentration of reactive nitrogen and oxygen species. Only the tap water activated by the plasma jet has shown a significant effect on the plant growth. This effect could be correlated to reactive nitrogen species such as nitrite/nitrate species present in plasma activated tap water.

## Introduction

Low temperature plasmas are initially composed by a background gas medium subjected to an electric or electromagnetic stress that allows to initial seed electrons (naturally present in the gas medium) to store energy high enough to undergo many inelastic processes with atoms or molecules of the background gas. Those inelastic ionization or dissociation or excitation collisions due to the energetic electrons lead to the creation of new plasma species as radicals, ions, long-lived excited species, neutral byproducts, photons and even electric field self-induced by the space charges present in the plasma. The physico-chemical properties of these active plasma species are exploited in many fields as for instance biomedical and plant biology.

Indeed, most research on the effects of plasmas on organisms began about a decade ago and has been conducted by physicists as well as by biologists. Systems studied include, for instance, wound healing or malignant cell inactivation (e.g. [1–3] and the references given therein). Prior to these investigations in this “plasma medicine” field, there is a long list of research devoted to sterilization and decontamination of surfaces based on the bactericide properties of low temperature plasmas. Most of this literature is devoted to the inactivation of planktonic microorganisms [4, 5] and some to biofilm removal [6, 7]. There is also research in the field of engineering of biomaterial and tissues exploiting, for instance, the ability of low temperature plasmas to change the surface properties and to functionalize surfaces [8, 9]. There are also studies in the field of plasma gene transfection which is promising for applications in regenerative medicine and/or gene therapy [10, 11].

However, the use of low temperature plasmas in the field of plant biology has been less investigated than in the biomedical field. The first studies date back to the 1990s where a patented glow discharge device using different gases at low pressure (O_2_ and N_2_-O_2_ mixtures) stimulated the germination and the growth of soybean seeds [12]. This was followed by a few studies devoted to plasma treatments of for instance the stimulation of germination of certain grain crops by using a low pressure direct-current and pulsed glow air discharges [13,14]. Another study demonstrated that low pressure helium radio-frequency discharge induces growth of tomato seeds and resistance to tomato wilt (a bacterial disease) after harvest [15]. There are also recent investigations on the effect of two nano-pulsed electric discharge devices on the stimulation of germination of brown mustard seeds [16]. In fact, seeds from various species (maize, oat, wheat, radish, tomato, bean, lentil, sunflower, honey clover and soy, etc.) were already studied up to now using different plasma setups (radio-frequency discharges, microwave discharges, gliding discharges, surface discharges, etc.) at low or atmospheric pressures using various gas compositions (Air, Ar, He, O_2_, or mixtures of part of them, etc.). However, only a very few of them are devoted to analysis on the plasma effects and the action mechanisms at the cellular level (see e.g. [17]).

Thus, even though low temperature plasmas commonly enhance germination and plant growth, the mechanism for this effect remains largely unexplored.

Here, we use *Arabidopsis thaliana*, a plant model for many years, to continue a previous studies on how low temperature plasmas affect germination that allowed us more particularly to select the plasma devices and the seed exposure times [18]. Here, we compare two plasma devices: (i) a helium plasma jet setup [19] and (ii) a floating electrode dielectric barrier discharge in ambient air [20] both powered by a pulsed high voltage generator.

It is noteworthy that seeds have generally important resistant structures necessary to ensure the transition between two successive generations. The formation of mature seed of *A*. *thaliana* takes about twenty days following fertilization. Seeds comprise three principal tissues that are, starting from outside to inside: (i) the testa or the seed coat; (ii) the endosperm, which is divided in 3 parts: the peripheral, the chalazal, and the micropylar endosperm, the last being adjacent to the radicle and; (iii) the embryo, enclosed by the two first envelopes, which comprises hypocoyl, cotyledon, and radicle [21].

Furthermore, plants produce endogenous reactive oxygen species such as hydrogen peroxide, superoxide, hydroxyl radicals, constitutively throughout their lives. Reactive oxygen species have a dual role and a dose-dependent effect [22]. At high concentrations, these species have an important oxidizing power and harm the development of the plant [15]. However, with a lower concentration, reactive oxygen species act as secondary messengers within the plant [23]. They are also involved in the regulation of *A*. *thaliana* seed dormancy [24] and in the breakdown of embryonic cell layers, such as testa rupture and endosperm rupture [25, 26]. It is noteworthy that the plants possess a battery of enzymes to achieve homeostasis for reactive oxygen species [22]. Class III peroxidases are part of these proteins; they can oxidize various phenolic compounds in presence of hydrogen peroxide and also regulated hydrogen peroxide concentration [27]. Class III peroxidases and hydrogen peroxide are co-localized in the testa and endosperm prior to rupture of these two envelopes [28]. More specifically, class III peroxidases are located at the periphery of the cells. The enzymatic activity of these proteins could play a role in the breakdown of the two cell envelopes to facilitate the radicle protrusion [29].

The present work consists of performing germination assays to confirm a positive plasma effect, and more precisely, the effects of plasma on the testa rupture and endosperm rupture since they are the two protective layers of the plant embryo. In addition, in order to study the long-term effect of plasma on plant development and growth, plasma-activated water is used as the source of water.

To access plasma-induced germination and effects of plasma-activated water on plant growth, we analyzed of reactive oxygen species and peroxidase activity based on colorimetric tests and we also analyzed seed membrane permeability. We used scanning electron microscopy (SEM) to underline the change on the seed surface after plasma treatment. Furthermore, to emphasize the role of long-lived aqueous reactive species on plant growth, we determined concentrations of protons, hydrogen peroxide, reactive nitrogen species and also conductivity of plasma-activated waters by using either distilled water or tap water.

## Materials and methods

### Plant material and biological assays

*Arabidopsis thaliana* (L.) Heynh of the Columbia ecotype (Col) was used throughout. For each germination test, between 150 seeds to 300 seeds were exposed to the low temperature plasmas. Germination assays were performed after the plasma exposure at various times t_i_ ranging from immediately at time t_0_ to times t_2_ = 2 days, t_7_ = 7 days and t_9_ = 9 days. Culture took place in 35 mm diameter Petri dishes containing Whatman paper circles (3 MM Chr Chromatography Paper: Medium thickness: 0.34 mm). The paper was soaked with 0.5 mL of distilled water, with the exception of boxes containing plasma-activated water or non-activated water. The boxes were sealed with ANAPORE surgical tape. Germination took place in a culture chamber under controlled conditions (40% humidity, temperature 22°C/20°C, photoperiod 16 h/8 h). The germination was analyzed 24 h and 40 h after imbibition. The Petri dishes were observed at the Toulouse imaging platform by using a Zeiss Axiozoom V16 stereomicroscope and the pictures were taken using a Zeiss HRC colour camera. Germination stages were distinguished, as follows: (i) the absence of rupture, (ii) testa rupture and (iii) endosperm rupture. Six to nine hundred seeds from two technical replicates and from three independent biological experiments were analyzed for each condition.

To measure the effect of the plasma-activated water on development, 40 *A*. *thaliana* plants were sown and cultivated in a growth room (75% humidity, temperature 24°C/20 °C, photoperiod 16 h/8 h). These plants were arbitrarily divided into four groups so that each one was watered with a particular kind of water: plasma activated distilled water, non-activated distilled water, plasma activated tap water and non-activated tap water. Watering was performed every 2 days using controlled volumes. The volume for watering was increased as plants develop (from 10 to 30 mL per week). Pictures of each plant were taken at regular intervals in order to perform phenotypic analysis (the number of leaves, the leaf area and the rosette diameter). Ten plants per assay were analyzed and this experiment was performed twice independently.

### Reactive oxygen species and nitrate quantifications; class III peroxidase activity and mucilage detection

Potassium permanganate (KMnO_**4**_, Sigma Aldrich and Saint-Quentin Fallavier, France) was used to quantify the concentration of hydrogen peroxide generated in the plasma-activated water. The assay of 1 mL of water or plasma-activated water was titrated with 20 mM of potassium permanganate and the staining was quantified thanks to the change of colour to the equivalence solution. Colour change occurred when concentration equivalence was reached between hydrogen peroxide and potassium permanganate.

Nitro Blue tetrazolium chloride (NBT, Sigma-Aldrich) was used to detect in the seeds the presence of superoxide ion. The seeds were covered with a 2 mM aqueous solution and incubated for 30 min at room temperature in the dark. A blue colour appears at the surface of the seeds when superoxide ion is present [30].

To detect the amount of nitrate in water, 1-phenol-2,4-disulfonic acid (25% C_6_H_6_O_7_S_2_ in sulfuric acid, Sordalab, Etampes, France) was used. The assay was carried out in a 96-well plate using 100 μL of 10X-concentrated sample in a final volume of 200 μL containing phenol-disulfonic acid and NaOH at 10 M (to obtain a basic medium). Absorbance was quantified at 405 nm.

To observe the activity of peroxidases, seeds were incubated at room temperature during 30 min in the dark in 1 mL of 200 mM phosphate buffer, pH 6.1, containing 0.125% guaiacol (Fluka-Sigma-Aldrich, Steinheim, Switzerland) and 150 μL of 11 mM hydrogen peroxide. The oxidation of the guaiacol by the peroxidase releases a brown precipitate.

Mucilage released by the seed was observed after incubation of the treated and control seeds with a solution of 0.2% ruthenium red (Sigma-Aldrich).

The pictures for the 3,3′-diaminobenzidine (DAB) staining, class III peroxidase activity and released mucilage were taken without seed checking to avoid the dispersion of the non-adherent mucilage layer by using a Zeiss Axiozoom V16 stereomicroscope and a Zeiss HRC colour camera.

### Membrane permeability test

The seed permeability assay was performed using 2,3–5 triphenyltetrazolium chloride (Tetrazolium red, Sigma Aldrich) that has the property to be reduced when it crosses the plasma membrane and form formazan, a red precipitate. Control seeds and seeds treated with the low temperature plasmas were incubated with a solution of 1% tetrazolium red for 40 h at 28°C. The seeds were rinsed with distilled water and ground in 1.5 mL of 95% ethanol. After centrifugation at 3500 rpm for 3 min, the formazan contained in the supernatant was assessed by measuring absorbance at 492 nm.

### Scanning electron microscopy (SEM)

The surface morphology and integrity of seeds were observed with SEM. Three independent batches involving treated and non-treated seeds were prepared. Each sample of 30 seeds was affixed to the surface of an aluminum stub with double-sided carbon tape and sputter coated with ~10 nm of platinum. SEM was carried out on a Quanta 250 FEG (FEI Company) at 5 kV acceleration voltage, a spot size of 3.0 and, a pressure of 3.50 x 10^−4^ Pa.

### Statistical analysis

Image processing was performed using ImageJ version 1.46r [31]. The results were analyzed statistically using software R studio version 1.0.136 (cran.r-project.org). Student’s tests and Wilcoxon tests were carried out to verify the normality of the results and the possible differences of the plasma effects between the different plasma treatments.

### Low temperature plasma devices for seed treatment and water activation

**Fig 1:**
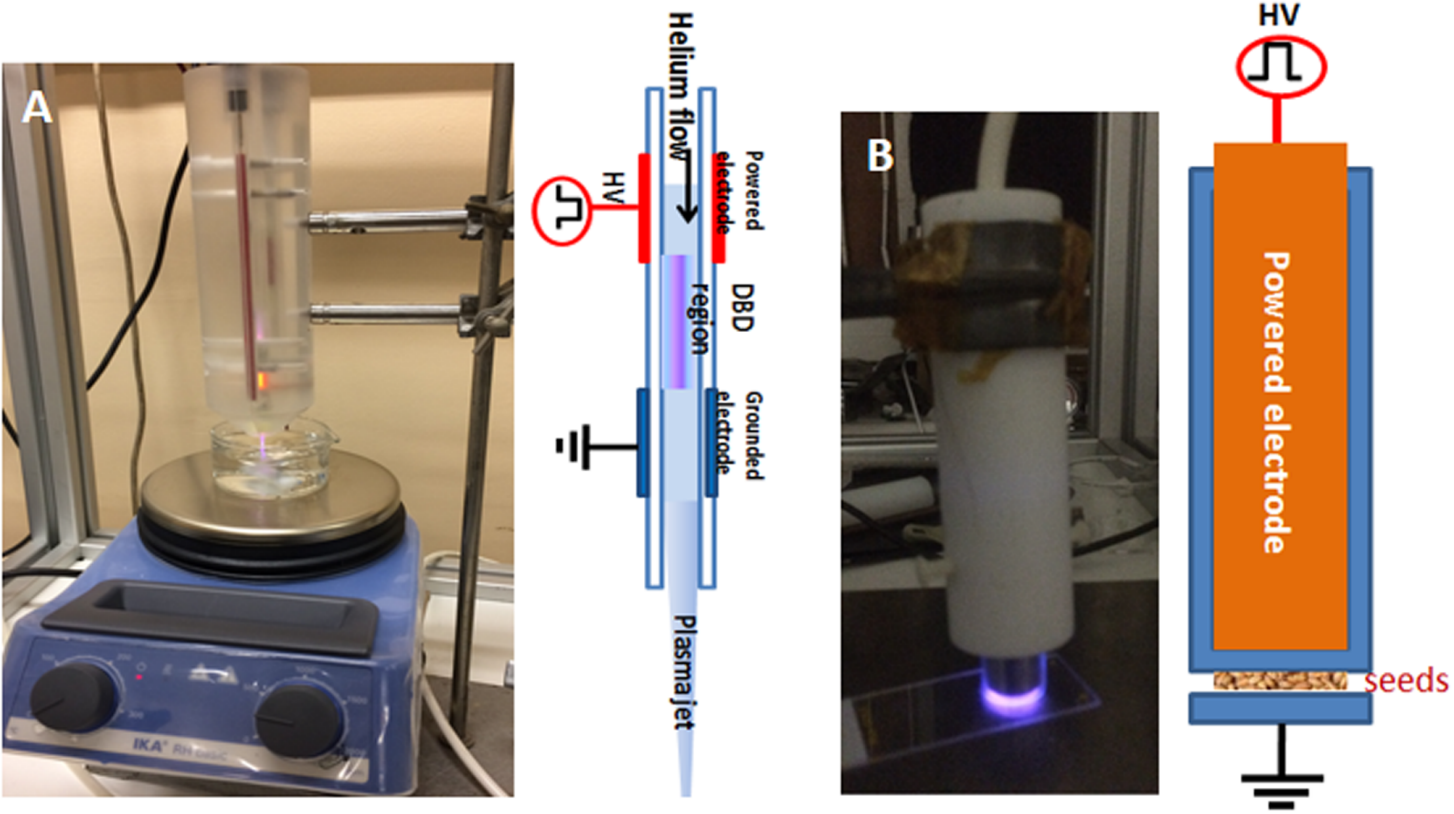
Different plasma devices. A. Schematic view of the helium plasma jet device on the right side and picture during the generation of plasma activated water inside a glass beaker using the helium plasma jet and a magnetic stirrer. B. Schematic view of the floating electrode-dielectric barrier discharges (FE-DBD) in air showing the powered electrode covered by glass (in blue colour) with seeds in ambient air plasma above the lamella (right side) and picture displaying the air plasma over the glass lamella.

For this study, two devices were used to generate low temperature plasmas generated at atmospheric pressure. The first device generates a plasma jet through a glass tube crossed by helium gas flow at atmospheric pressure [19]. This helium plasma jet launched in ambient air was used in both direct seed treatment and the water activation (Fig 1A). The second device is a dielectric barrier discharge generated in ambient air without rare gases and uses the seeds as a floating electrode [20] (Fig 1B). We refer to it here as: floating electrode dielectric-barrier discharge (FE-DBD).

The parameters of the pulsed power supply used to generate these two low temperature plasmas are 10 kV for the voltage, 9.7 kHz for the frequency and 1 μs for the pulse duration.

In the case of the plasma jet, the helium (with 4.5 for purity) was injected at 3 L min^-1^ through a glass tube (4 mm inner diameter). The distance between the tube outlet and the treated target (either seeds or water) was fixed at 2 cm.

Two types of treatment were performed with the helium plasma jet.

1. For the direct treatment, 200 to 300 *A*. *thaliana* seeds are placed in a specific Eppendorf tube with a round bottom. The seeds were stirred half way through the exposure time to ensure a better homogeneity of the plasma treatment. A control test was carried out with a plasma-free helium flow. The optimal exposure time of 15 min was based on previous work [18].
2. For the water activation by the plasma jet, 30 mL of water, inside a beaker with magnetic stirring, were exposed to the plasma jet for 15 min. This activates distilled water (PAW_H2Od_) and tap water (PAW_tap_). To reduce experimental bias, the water volume needed for the 5 weeks involving germination and plant growth was set aside and stored at 4°C in a large bottle at the initial time t_0_. Then the required water volume per week (from 150 to 300 mL) was activated by the helium plasma jet at the beginning of each week. Controls with non-activated water were also executed. The plasma-activated water was stored at 4 °C in the dark to avoid the loss of reactive nitrogen and oxygen species.

In the case of the floating electrode dielectric-barrier discharge setup, the plasma formation is substantially different from the helium plasma jet since the plasma is generated directly in ambient air without using rare gases. The cylindrical high-voltage electrode, 8 mm in diameter, is covered by a glass dielectric with practically 8 mm for the inner diameter (Fig 1B). The grounded electrode is a thin glass plate (1 mm thickness) on which the seeds are placed. Six hundred to 800 *A*. *thaliana* seeds arranged on this glass plate in a circular manner (diameter of about 8 mm), are directly exposed to the air plasma action. The generated plasma species (radicals, excited, charged particles and photons) with the associated space charge electric field necessarily leads to a better plasma energy and efficiency than the case of helium plasma jet setup [20].

## Results

### Low temperature plasmas stimulate *A*. *thaliana* germination

**Fig 2:**
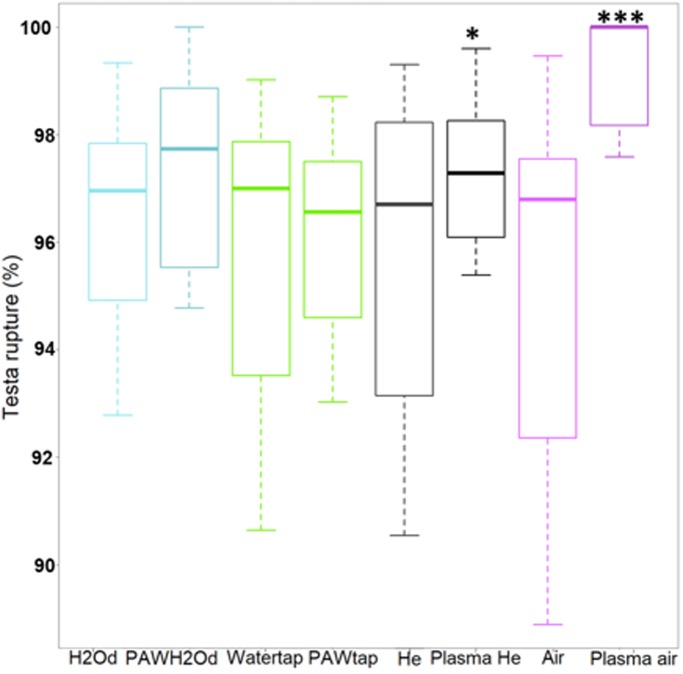
Direct and indirect plasma effects on germination rate of *Arabidopsis thaliana* seeds. The boxplots show the effects of different applied plasmas on the testa rupture. Observations are made on four independent assays at t0+24h. Six hundred seeds are counted for each batch. Wilcoxon test has been used for the significance between the means, the p-value is: * < 0.05; ***<0.002 (For data see S1 Fig).

Here we tested the effects of *Arabidopsis thaliana* germination rate of direct plasma treatment (helium plasma jet and air FE-DBD) and the indirect plasma actions (plasma activated tap and deionized waters). The seed germination rates were first determined for imbibition immediately after the plasma treatment (at time labelled t_0_). Seeds treated with the air plasma had a significant increase of testa rupture and endosperm rupture 24 h and after imbibition when compared to the non-treated seeds (Fig 2). For the helium plasma jet, the only significant effect was observed 24 h after imbibition with an increase of the testa rupture (p-value = 0.01068). No significant effect was observed when the seeds were imbibibed with any of the plasma activated waters (Fig 2).

To determine whether the plasma treatment effect was retained, we assayed germination when imbibition followed the plasma treatment by 2, 7, or 9 days. As the air plasma effect appeared to be the strongest with immediate imbibition, the air plasma device was used for this test.

**Fig 3:**
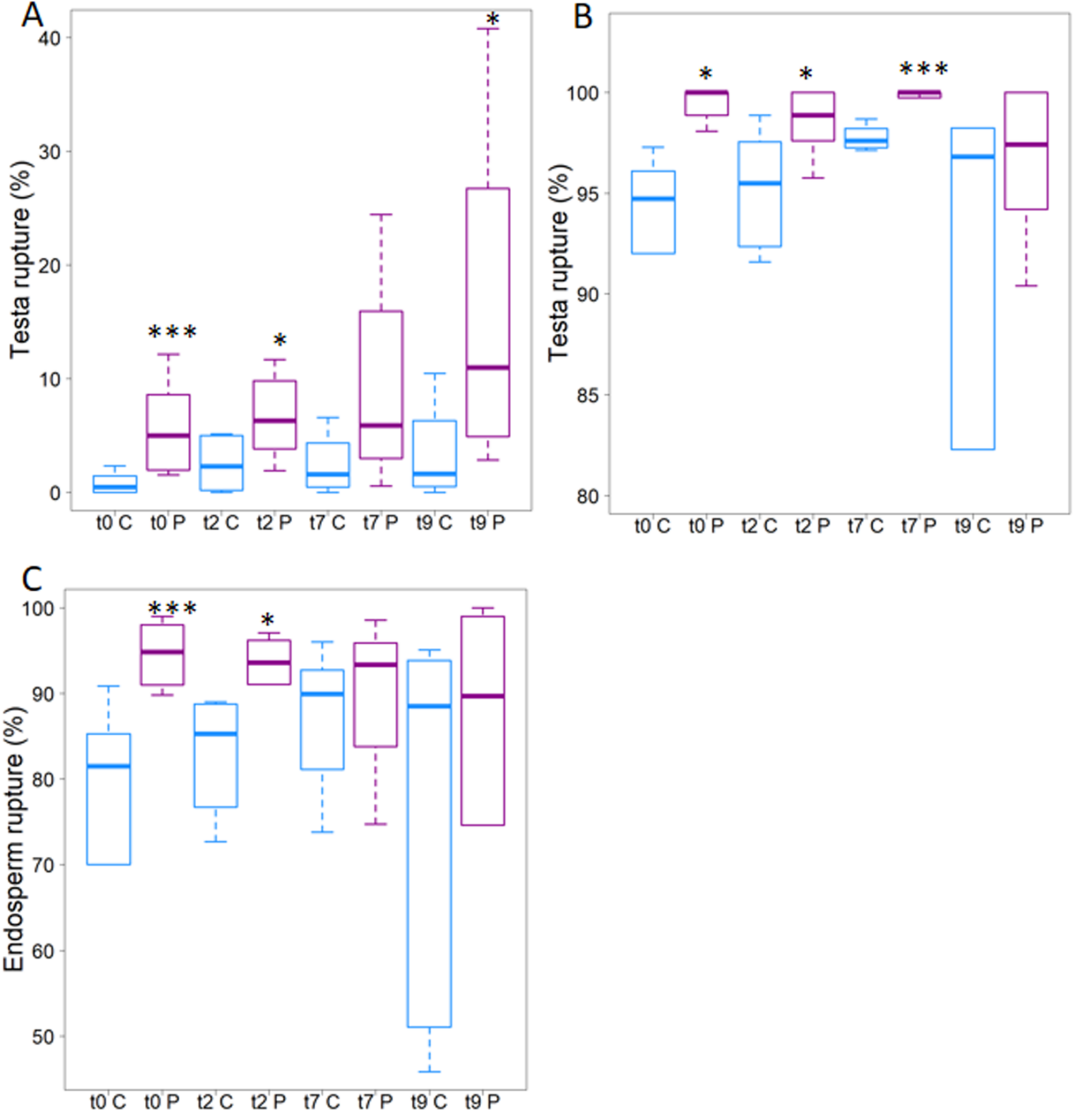
Air plasma stimulated germination rate of *A*. *thaliana*. Boxplots show control (cyan) and treated (magenta). A. Testa rupture as a function of time between treatment and imbibition (times t_0_, t_2_, t_7_ and t_9_ are given in days), observed 24 h after imbibition. B. As panel A but observations made at 40 h after imbibition. C. Endosperm rupture (observations at 40 h). Six hundred seeds are counted for each of the four considered batches. Wilcoxon test has been used to test the significance between the means, the p-value is: * < 0.05; *** < 0.002 (For data see S1 Fig).

We assayed testa rupture at 24 h (Fig 3A) and 40 h (Fig 3B) and endosperm rupture after 40 h. For testa rupture at 24 h, the plasma treated increased the incidence of rupture from 1% or 2% in the controls to about 5%. The effect was highly significant with imbibition immediately after plasma treatment and became less so as time increased between plasma treatment and imbibition, although this lessened significance appeared to result from greater variability in the samples, both control and plasma-treated. At 40 h after imbibition, the plasma treatment increased the incidence of testa rupture from around 95% to nearly 100%, with the effect becomes less clear with a 9 days interval again owing to the variability among the seeds (Fig 3B). These trends were confirmed by scoring endosperm rupture (Fig 3C).

**Fig 4:**
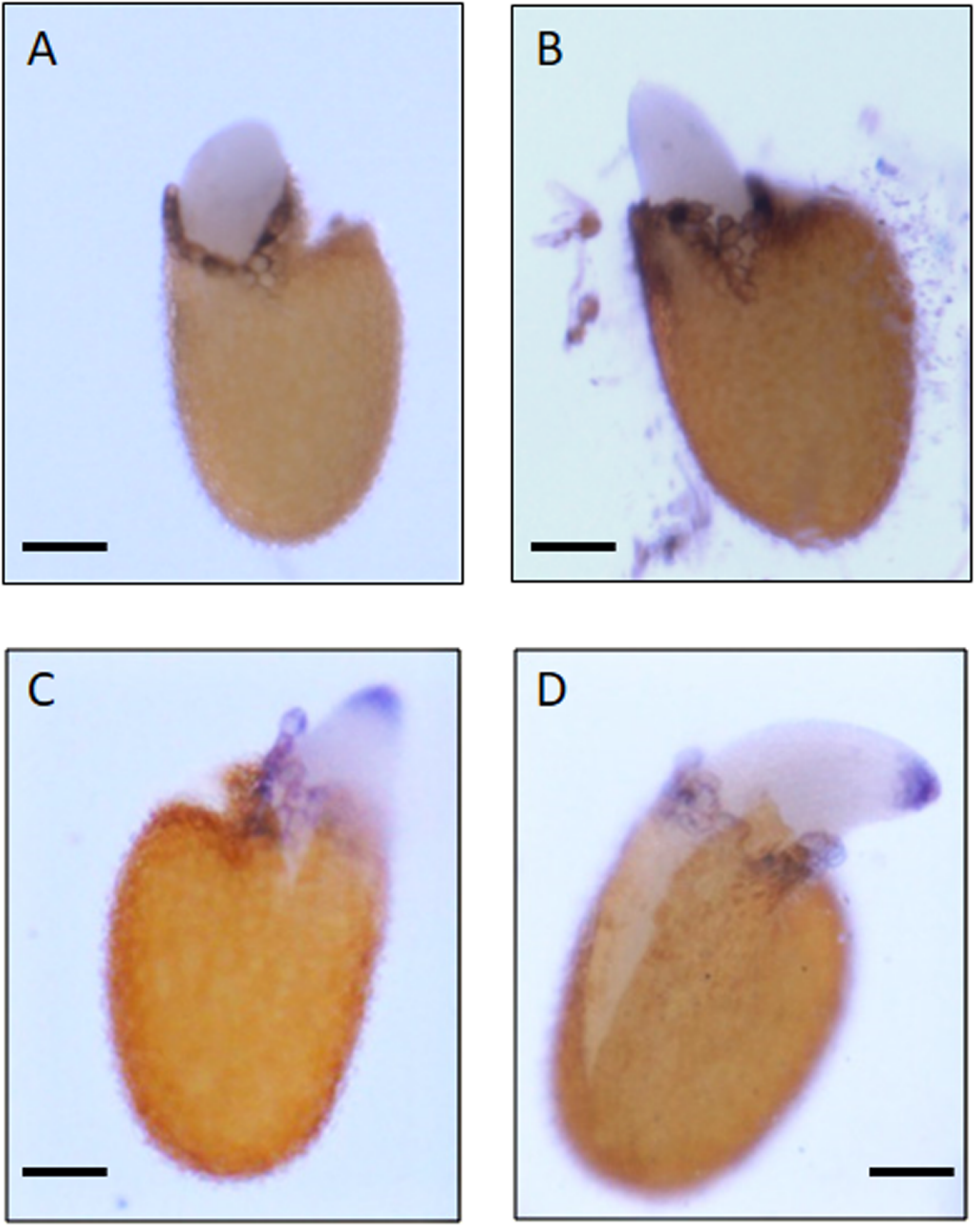
Class III peroxidases activity and reactive oxygen species detection. Class III peroxidases activity was detected seeds by using guaiacol as substrate (A and B). Superoxide ion O_2_^.-^ was observed y with NBT (C and D). Control seeds (A and C) and seeds treated with air plasma (B and D). The intensity of the coloration appeared to be identical at the same level of germination for both cases (control or plasma treated seeds). Each scale bar corresponds to 100 μm.

Finally, Figs 2 and 3 show an increase of testa and endosperm rupture incidence for seeds treated by the air plasma as compared to control seeds. The early stages of germination (e.g. testa and endosperm ruptures) are generally controlled in part by reactive oxygen species. As the plasmas are a known source of reactive oxygen species, we analyzed plasma treated seeds for evidence of these species. As expected, class III peroxidases activity, as well as superoxide, was detected in the cells neighboring the rupture region (Fig 4 and [28]) but in terms of quantity no difference was observed between the treated and non-treated seeds. Due to the increased germination rate, stained seeds were observed after plasma treatment regarding class III peroxidases activity as well as superoxide detection. This indicates that the plasma treatment did not modify the quantity of produced reactive oxygen species but could accelerate their productions.

**Fig 5:**
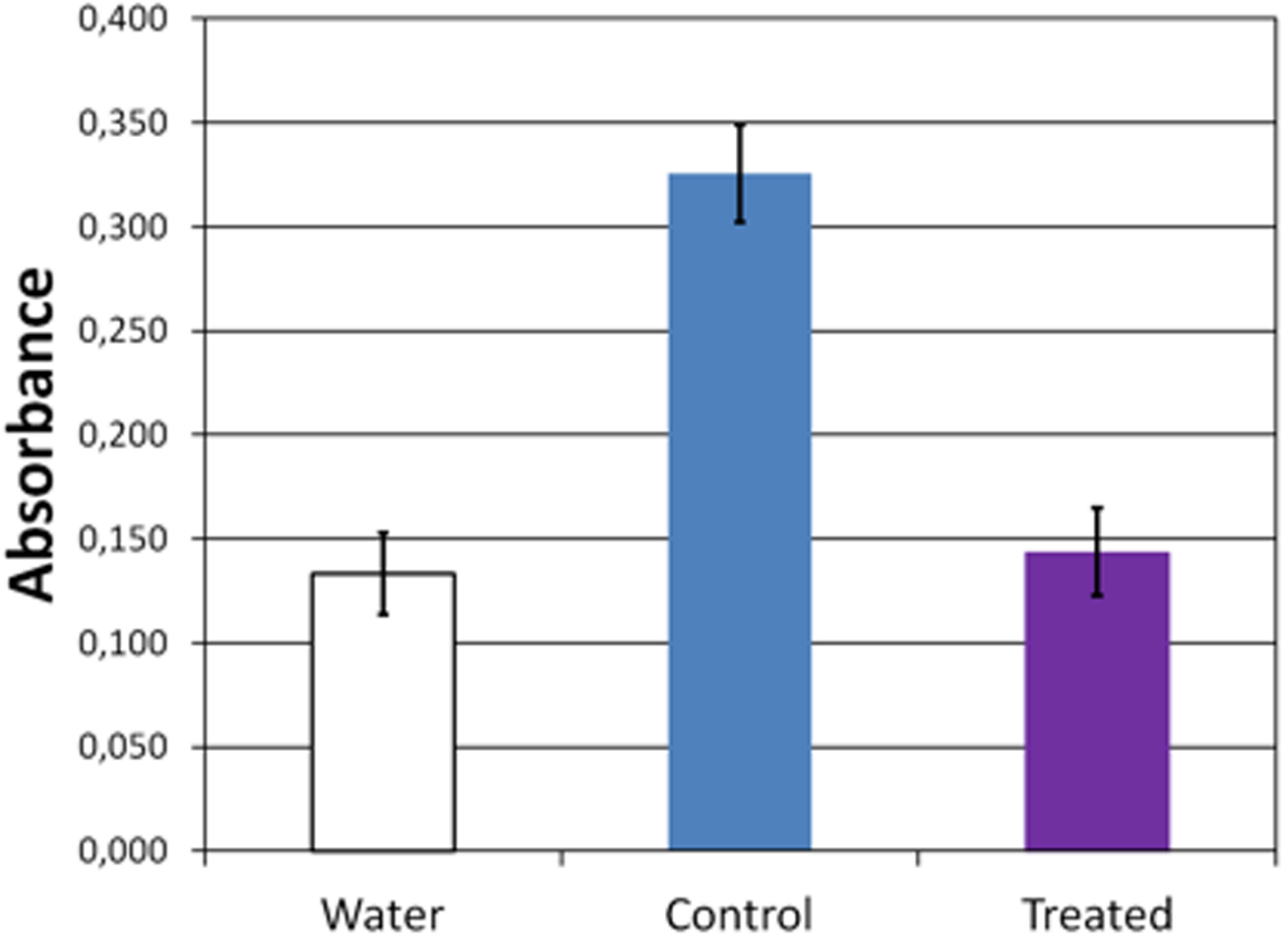
*A*. *thaliana* seed permeability assay. Seeds were incubated for 24 h in tetrazolium red at 28°C and formazan production assayed by measuring absorbance of the incubation solution at 492 nm. For plasma treated (purple bar), the air plasma was used and seeds were incubated with tetrazolium immediately following treatment. Control (blue bar) were treated similarly but without activating the plasma. Water (white bar) treatment omitted the tetrazolium. Bars show mean of 4 assays and the error bars are 0.05 (For data see S1 Fig).

Seed imbibition is one of major factors controlling the transition from dry seed to germinating seed. Such as most of the plant aerial organs, *A*. *Thaliana* seeds possesses hydrophobic layer (cuticle) containing fatty acid. Recent studies have demonstrated that decrease of fatty acid containing alcohols and diols functional groups in the seed, both increase the seed coat permeability and the seed germination sensitivity to abscisic acid [31] and to gibberellin [32]. The permeability of *A*. *thaliana* seed coat is strongly enhanced in seeds with defect in fatty alcohols and diols [31, 33]. The treatment with the air plasma seems to largely modify the seed coat permeability as treated seed produced little if any red formazan is detected (Fig 5). This modification might be responsible for the variation in germination rate. Recent studies performed on pea seeds have demonstrated an acceleration in water uptake following treatment with low temperature plasmas [34].

**Fig 6:**
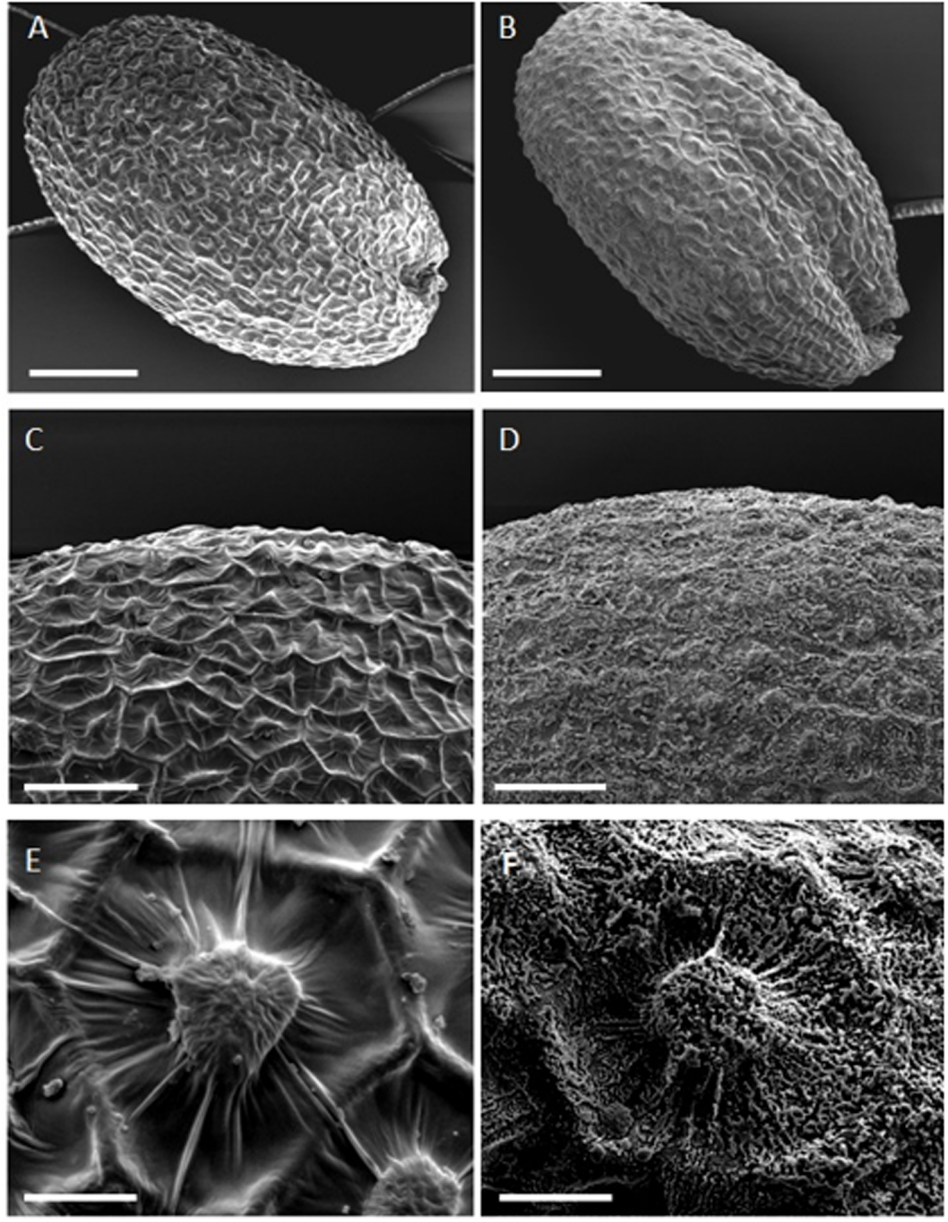
Scanning electron microscopy (SEM) images of *A*. *thaliana* seeds. (A, C, E) are the control while (B, D, F) are the air plasma treated. Scale bars: 100 μm A, B; 50 μm C, D; 10 μm E, F.

The seed coat has a major function during early germination steps, acting both as a physical protection against environment and as a constraint for radicle protrusion. Therefore any structural modification of this envelope could affect seed germination. To examine the seed coat, we used SEM. The SEM images show that plasma treatment reduced the prominence of the volcano-like protuberances (Fig 6A–6D) and changed the appearance of the surface of the seed, making it more granulated (Fig 6C–6F). The alteration of the seed surface due to plasma exposure can be associated with a modification of seed coat water uptake and therefore might directly affect the germination rate of the plasma treated seeds. Indeed, an increase of the germination rate and a change in the surface structure has previously been reported for safflower (*Carthamus tinctorium*) and pea seeds [34, 35].

In addition, the modification of the seed coat surface is not transitory insofar as we observed similar changes of the seed surface 7 days after the plasma treatment.

### Plasma-activated water stimulates *A*. *thaliana* development

**Fig 7:**
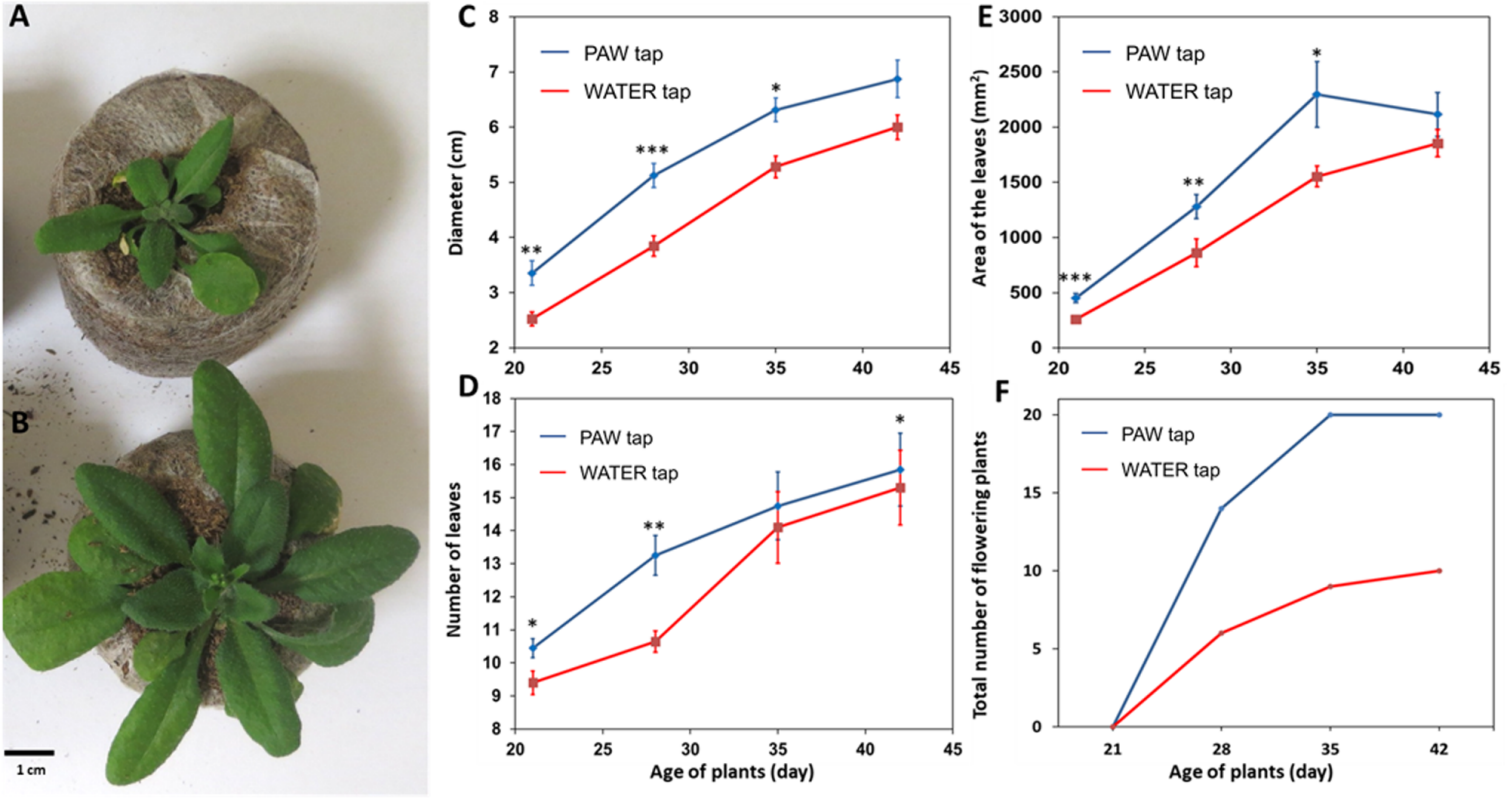
Effects of plasma-activated tap water (PAW_tap_) on *A*. *thaliana* growth. Photos after 28 days of development watered by tap water (A) and PAW_tap_ (B). Diameter of the rosette (C); *A*. *thaliana* seedling leaf number (D); area of the leaves (E), and total number of flowering plants (F). Wilcoxon test was used to test the significance between the means with p-value indicated as: · < 0.1; *<0.05; **<0.005; ***<0.002 (For data see S1 Fig).

In parallel to the germination assays, we tested the effect of plasma activated water on plant growth. Deionized and tap waters were exposed to the helium plasma jet for 15 min. Then the *A*. *thaliana* plants were regularly watered using four kinds of waters (deionized water, plasma-activated deionized water, tap water and plasma-activated tap water) during their growth until their final stage, reached after 42 days. It is noteworthy that only plasma-activated tap water had a significant effect on the plant growth while the use of plasma-activated deionized water give similar results as the control cases watered either using deionized water or tap water (data not shown). Then, various plant parameters such leaf number, rosette diameter, leaf area, and total number of flowering plants were quantified (Fig 7C–7F). All the measured parameters are significantly increased when using plasma-activated tap water.

Regarding the diameter of the rosette, a significant increase was observed when using the plasma activated-water (Fig 7C). At 21 days, there was an increase of 0.83 cm; at 28 days the increase was about of 1.28 cm; finally at 35 days, the average diameter of the plants watered with plasma-activated tap was still about a 1 cm larger than the controls.

Regarding the number of leaves, a significant effect was observed at 21 days after sowing, with an average increase of the treated plants compared with the control of about one leaf per plant (Fig 7D). There is also a positive effect of plasma-activated water at 28 days.

Furthermore regarding the leaf area, there was a significant increase visible at the first three observation times (Fig 7E). At the first time (21 days), there was an increase of about 200 mm^2^. At the second time (28 days) the increase was equal to about 400 mm^2^ while at the third observation time (35 days) the increase reached about 750 mm^2^.

The development of the inflorescence stem showed that there were more plants with flowers at all development stages when the plasma-activated tap water was used for the watering (Fig 7F). We also noted that all the plants had flowered by 35 days when using plasma-activated tap water while it was not the case of the plants watered with the tap water.

For a better understanding of the plant growth stimulation by plasma activated water, some chemical characteristics of the tested waters were analyzed (Table 1). As expected, hydrogen peroxide was undetectable in the non-activated waters (Water_tap_ or H_2_O_d_) while significant concentrations of hydrogen peroxide were detected in plasma-activated waters (Table 1).

**Table 1:** Physico-chemical parameters of waters. Electric and chemical parameters of deionized water (H_2_Od), tap water (Water_tap_) and plasma-activated deionized water (PAW_H_2_Od_) and tap water (PAW_tap_). The conductivity is expressed in relative value differences: Δσ/σ. The measurements of concentrations, pH and conductivity are made 3 times independently.

Water	H_2_O_2_ concentration (mg.L^-1^)	NO_3_^-^ concentration (mg.L^-1^)	Variation of electrical conductivity Δσ/σ	pH
H_2_Od	0	0	Δσ H_2_O_2_d	7.41 ± 0.13
PAW_H_2_Od_	17.0 ± 0.1	0	20 x σ H_2_O_2_d	7.07 ± 0.04
Water_tap_	0	5 ± 2	σ Water_tap_	8.02 ± 0.25
PAW_tap_	25.5 ± 0.1	15 ± 2	2 x σ Water_tap_	7.62 ± 0.13

The nitrate ion concentration was estimated by using an assay based on the reaction between the nitrate ion and the aromatic ring of 1-phenol-2,4-disulfonic acid. As expected, the concentration of nitrate ions was significantly increased (3 times bigger) in the tap water when it was activated by the plasma while it is negligible in deionized waters (activated or not).

The increase of the electrical conductivity when the water (deionized or tap) is activated by the low temperature plasma was consistent with results in the literature [36]. The plasma activation of the water also changed acidity. The presence of a larger concentration of these reactive nitrogen ions in the plasma-activated tap water could partly explain the faster development of the plant when it is watered with this plasma-activated water.

## Discussion

The significant increase of germination rate of *A*. *thaliana* seeds observed after plasma air treatment is due neither to a modification of the mucilage release nor to a global change of production of reactive oxygen species. However, the early reactive oxygen species production was certainly favoured in the case of plasma treated seeds. Indeed, exogenous addition of reactive oxygen species largely speed up the first steps of germination [28] as observed with plasma treated seeds. On the other hand, major modification of the seed surface and a reduction of testa permeability were observed. This appeared contradictory to a previous study which showed an increase of water uptake for pea seed treated with plasma [34]. This discrepancy might be related to the difficulty of performing water uptake with *A*. *thaliana* seeds, which have the capacity to extrude a mucilage layer in presence of water and which are much smaller than pea seeds.

SEM has shown new and lumpy structures on the seed surface after plasma treatment. This needs to be further analysed to better understand the mechanisms of germination stimulation induced by low temperature plasmas. Independently of these structural changes, we cannot exclude the formation of new functional groups which could stimulate the seed germination [37].

Regarding the effect of the plasma activated water on the plant growth, both conductivity and hydrogen peroxide levels were increased in the two kind of plasma activated waters (tap and deionised waters). Furthermore, we observed a clear increase of nitrate concentration in the plasma activated tap water. Nevertheless, among the four considered waters only plasma activated tap water had a significant effect on the plant development. This certainly means that some long-lived aqueous reactive nitrogen species as nitrate ions are responsible of the observed efficient growth of the plants.

## Supporting information

S1 Fig Page 1 (Data for Fig 2): Percentages of testa rupture versus the different kinds of treatment: deonized water (H2Od), plasma activated deonized water (PAW H2Od), tap water, plasma-activated tap water (PAWtap), helium flow (He), Plasma Helium, ambiant air and Plasma air. Page 2 (Data for Fig 3): A. Percentages of testa rupture for control (C) and plasma (P) versus different days after treatment: 0 day (t0), 2days (t2), 7 days (t7) and 9 days (t9) and 24h after imbibition. B. Percentages of testa rupture for control (C) and plasma (P) versus different days after treatment: 0 day (t0), 2days (t2), 7 days (t7) and 9 days (t9) and 40h after imbibition. C. Percentages of endosperm rupture for control (C) and plasma (P) versus different days after treatment: 0 day (t0), 2days (t2), 7 days (t7) and 9 days (t9) and 40h after imbibition. Page 3 (Data for Fig 5): Absorbance for water, control and plasma treatment: 5 successive tests and their average. Page 4 (Data for Fig 7): C. Diameter of the rosette versus age using tap water and plasma-activated tap water. D. Number of leaves versus age using tap water and plasma-activated tap water. E. Area of the leaves versus age using tap water and plasma-activated tap water. F. Total number of flowering plants versus age using tap water and plasma-activated tap water.
(XLSX)
